# Case report: pathological complete response after S-1/oxaliplatin regimen combined with trastuzumab and tislelizumab in patients with locally advanced gastric cancer

**DOI:** 10.3389/fonc.2024.1425572

**Published:** 2024-09-05

**Authors:** Chenglou Zhu, Mingxu Da, Yaoqi Li, Lingzhi Peng

**Affiliations:** ^1^ The First School of Clinical Medicine, Lanzhou University, Lanzhou, China; ^2^ Department of Surgical Oncology, Gansu Provincial Hospital, Lanzhou, China

**Keywords:** advanced gastric cancer, SOX regimen, trastuzumab, tislelizumab, neoadjuvant chemotherapy

## Abstract

**Background:**

The efficacy of a regimen combining Tegafur, Gimeracil and Oteracil Potassium Capsules (S-1), oxaliplatin (SOX) with trastuzumab and tislelizumab chemotherapy for advanced gastric cancer (GC) has not been reported.

**Case summary:**

A 56-year-old male was diagnosed with GC combined with peripheral lymph node metastasis. The patient received neoadjuvant chemotherapy, including SOX, tislelizumab and trastuzumab. After 4 cycles of chemotherapy, the tumor shrank significantly, and radical surgery was performed with good clinical results. To date, the patient has been followed up for 6 months with no significant side effects.

**Conclusion:**

In this study, the patient received combination chemotherapy with SOX trastuzumab and tislelizumab and successfully underwent radical surgery with good clinical outcomes. Combined SOX with trastuzumab and tislelizumab may be an effective neoadjuvant chemotherapy regimen.

## Introduction

Gastric cancer (GC) is one of the most common malignant tumors of the digestive tract, ranking third among cancer-related deaths in the world (1). China has a higher incidence of GC, with the third and second morbidity and mortality of malignant tumors, respectively (2). Surgery is still considered to be the only possible cure. Although the 5-year survival rate of early gastric cancer is more than 90%, more than 70% of the patients have developed advanced GC when they are diagnosed, and the prognosis is poor (3). With the rapid development of multidisciplinary treatment, neoadjuvant chemotherapy combined with R0 resection and lymph nodes dissection and postoperative radiotherapy or chemotherapy have become the main treatment modes for potentially resectable advanced GC, and have been proved to significantly improve the survival and prognosis (4, 5). Since Wilke et al (6) first reported the application of neoadjuvant chemotherapy in GC in 1989, neoadjuvant chemotherapy has become an important new method for the treatment of advanced GC. A large number of studies have shown that preoperative neoadjuvant therapy can effectively reduce tumor stage, increase R0 resection rate and improve overall survival of patients with advanced GC (7–10).

HER2-positive GC is a unique subtype of the disease, and its diagnosis and treatment strategy are different from those of HER2-negative GC. Patients with HER2-positive advanced GC can benefit from anti-HER2 therapy; however, there is currently no effective and standardized chemotherapy regimen for patients with HER2-negative advanced GC. Anti-PD-1/PD-L1 therapies have been approved for the treatment of advanced GC in clinical practice (11). A multicenter phase III randomized controlled trial (Checkmate649) proved that combined chemotherapy based on PD-1 inhibitor can effectively improve the overall survival rate (OS) and progression-free survival rate of patients with advanced GC compared with chemotherapy alone, and the adverse reactions are acceptable (12).

In the field of GC treatment, both perioperative chemotherapy regimens and first-line immune-combination chemotherapy regimens have demonstrated significant efficacy and safety. The RESONANCE phase III trial demonstrated that perioperative chemotherapy with tegafur, gimeracil, and oteracil potassium capsules (S-1) combined with oxaliplatin (SOX) significantly improved three-year progression-free survival by 7.9% (log-rank p=0.019) and increased the R0 resection rate by 11.2% (p<0.0001) compared to direct surgery, while maintaining tolerable toxicity (13). This combination regimen enhances patient prognosis. Meanwhile, the CheckMate 649 study established that in combination of nivolumab with chemotherapy significantly extended overall survival and improved quality of life in patients with advanced or metastatic non-HER2-positive GC. This underscores the growing role of immunotherapy in combination with chemotherapy for managing GC (14). Furthermore, a retrospective study demonstrated that trastuzumab enhanced the efficacy of chemotherapy combined with immunotherapy in patients with HER2-positive GC. This combination resulted in a significantly better objective remission rate and progression-free survival compared to chemotherapy alone, thereby reinforcing the critical role of trastuzumab in enhancing treatment outcomes (15).

In this study, the treatment scheme in this study was SOX combined with trastuzumab and tislelizumab, and then radical gastrectomy and D2 lymph node dissection were performed. Postoperative pathologically examination showed that the patient were in complete remission and no tumor cells were found, tumor regression grade (TRG) is grade 0, the neoadjuvant therapy was successful with good clinical effects. Before treatment, the patient agreed and signed an informed written consent form. This case report and related images were published, with informed written consent from the patient.

## Case presentation

### Chief complaints

A 56-year-old male was admitted to the hospital with acid reflux and belching discomfort for 6 months.

### History of present illness

Six months ago, the patient presented with acid reflux belching with no apparent cause, accompanied by tolerable vague upper abdominal pain with a burning pattern and one black stool. The abdominal pain has worsened over the past 2 months. The patient had no other clinical symptoms. The patient had lost 8 kg of weight since the onset of the disease.

### History of past illness

The patient had no previous medical history. There was no history of similar illness in the family.

### Physical examination

On physical examination, the abdomen was flat, and no gastrointestinal or peristaltic waves were observed. There was abdominal softness and deep tenderness in the upper abdomen. No rebound pain or muscle tension, non-palpable touch liver and spleen under the rib, and a negative Murphy sign were found. Shifting dullness was not present. Intestinal sounds were normal, and no abdominal vascular murmurs were heard.

### Laboratory examinations

The examination after admission showed an alpha fetoprotein (AFP) level of 3.92 ng/mL, CA125 level of 11.5 U/mL, CA199 level of 6.59 U/mL, carcinoembryonic antigen (CEA) level of 0.63 ng/mL, glycated antigen 72-4 (CA72-4) level of 3.16 U/mL, pepsinogen I (PGI) level of 21.9 ng/mL, pepsinogen II (PGII) level of 4.0 ng/mL, hemoglobin level of 16.4 g/L, and the fecal occult blood test was negative.

### Imaging examinations

**Figure 1:**
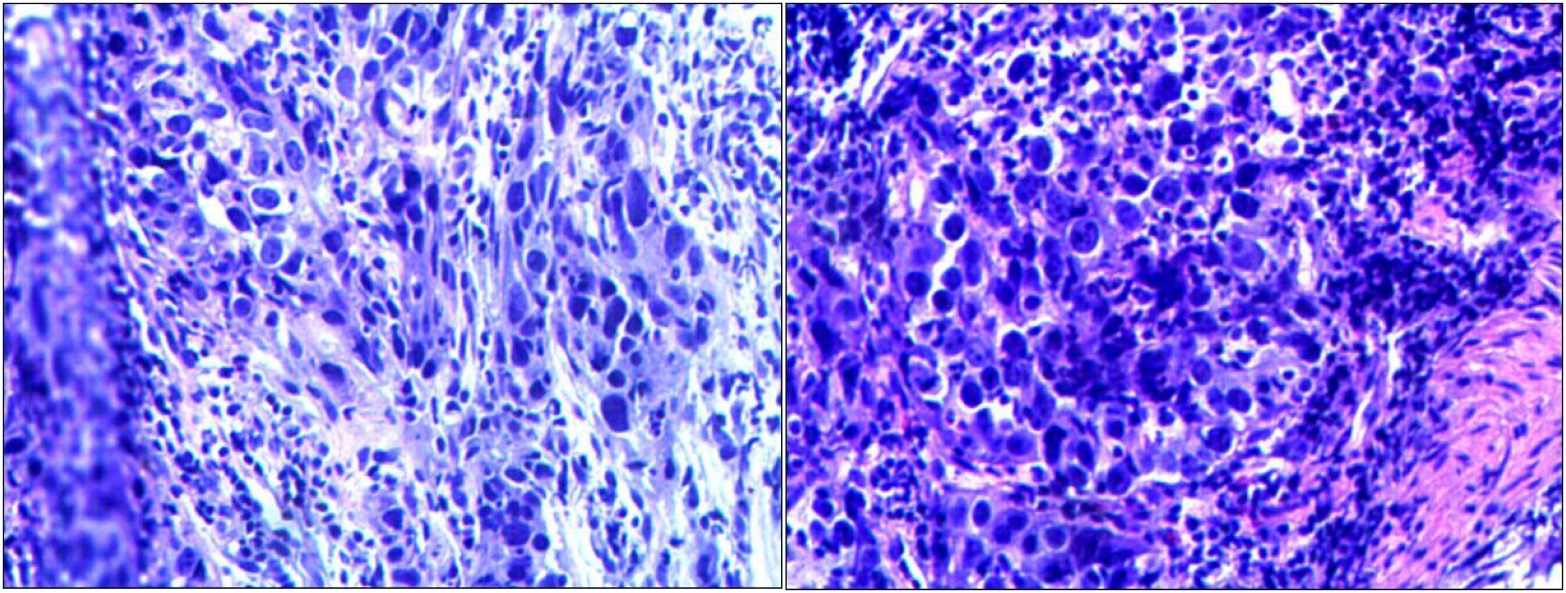
Pathological section: hypofractionated adenocarcinoma of the gastric horn, diffuse type.

**Figure 2:**
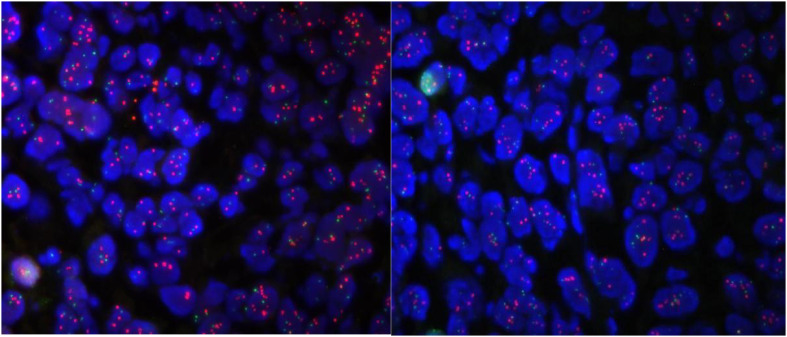
Molecular pathology diagnosis report: ① fluorescence microscopy is as follows, tumor heterogeneity is obvious, microscopic cancer area is visible ② FISH results: HER2 signal dotted distribution, counted tumor cells ≥ 20. mean copy number of HER2 8.05; mean copy number of CEP17 signal 3.45. HER2/CEP17 ratio = 2.33 > 2.2.

**Figure 3:**
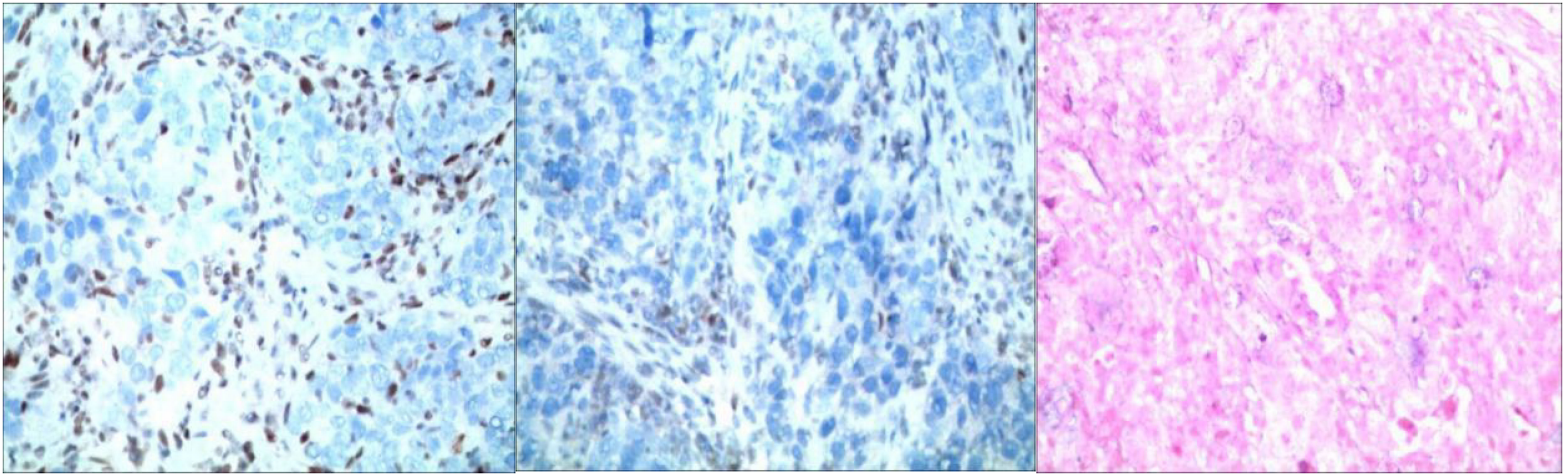
Immunohistochemistry: staining method: VENTANA fully automated immunohistochemical staining. Labeling results: MLH1 (-internal control +); MSH (+); MSH6 (+); PMS2 (-internal control +); EBER (-).

Routine gastroscopy revealed ulcers on the gastric antrum. The pathological diagnosis of endoscopic biopsy (**Figure 1**) showed poorly differentiated adenocarcinoma of the gastric antrum. Immunohistochemical analysis revealed positive HER2 expression (**Figure 2**), and immunohistochemical results were negative for MLH1 and PMS2 (**Figure 3**). The molecular pathology diagnostic test for microsatellite instability indicates that MSI-H microsatellites are highly unstable. Enhanced computed tomography (CT) revealed significant thickening of the gastric wall in the antrum. Enhanced CT revealed uneven enhancement, scattered small lymph nodes in the hepatogastric space. The clinical diagnosis was gastric antrum adenocarcinoma with lymph node metastasis, and the imaging stage was cT4aN1M0, Phase IIIa.

## Final diagnosis

The final diagnosis was gastric malignancy, with imaging staging of cT4aN1M0, which corresponds to stage IIIa. The pathologic diagnosis from the endoscopic biopsy revealed low-differentiated adenocarcinoma of the gastric antrum, classified as diffuse type according to Lauren staging. Immunohistochemical results indicated HER positivity, while MLH1 and PMS2 were negative. Additionally, molecular pathology testing demonstrated that the tumor exhibited high microsatellite instability (MSI-H).

## Treatment

The patient received treatment that included SOX, trastuzumab and tislelizumab. SOX consisted of an intravenous injection of oxaliplatin (iv) at 130 mg/m^2^ on day 1 and an oral 60 mg tegafur capsule twice a day (days 1–14), repeated every 3 weeks. Trastuzumab was administered at an initial dose of 8 mg/Kg, followed by 6 mg/Kg once every three weeks. The first infusion time is 90 minutes. If the patient tolerates the first infusion well, the duration of the subsequent infusion is changed to 30 min.Tislelizumab 200mg, administered every 3 weeks. Throughout the treatment process, the patients showed good tolerance to the chemotherapeutic drugs, did not experience any significant discomfort or serious side effects, had high treatment compliance, and were able to successfully complete each course of treatment as planned (**Table 1**).

**Table 1:** Timeline of Patient Care and Medication Dosing Schedule.

Date/Medication	Oxaliplatin (mg)	Tegafur (BID) (mg)	Tislelizumab (mg)	Trastuzumab (mg)
First (2021.02.22)	480	40	200	640
Second(2021.03.21)	480	40	200	450
Third(2021.04.16)	240	40	200	450
Fourth(2021.05.10)	240	40	200	450
Fifth (2021.06.07)	Radical distal gastrectomy, gastro-duodenal Billroth I anastomosis + D2 lymph node dissection			
Sixth(2021.07.05)	230	40	200	410

## Outcome and follow-up

**Figure 4:**
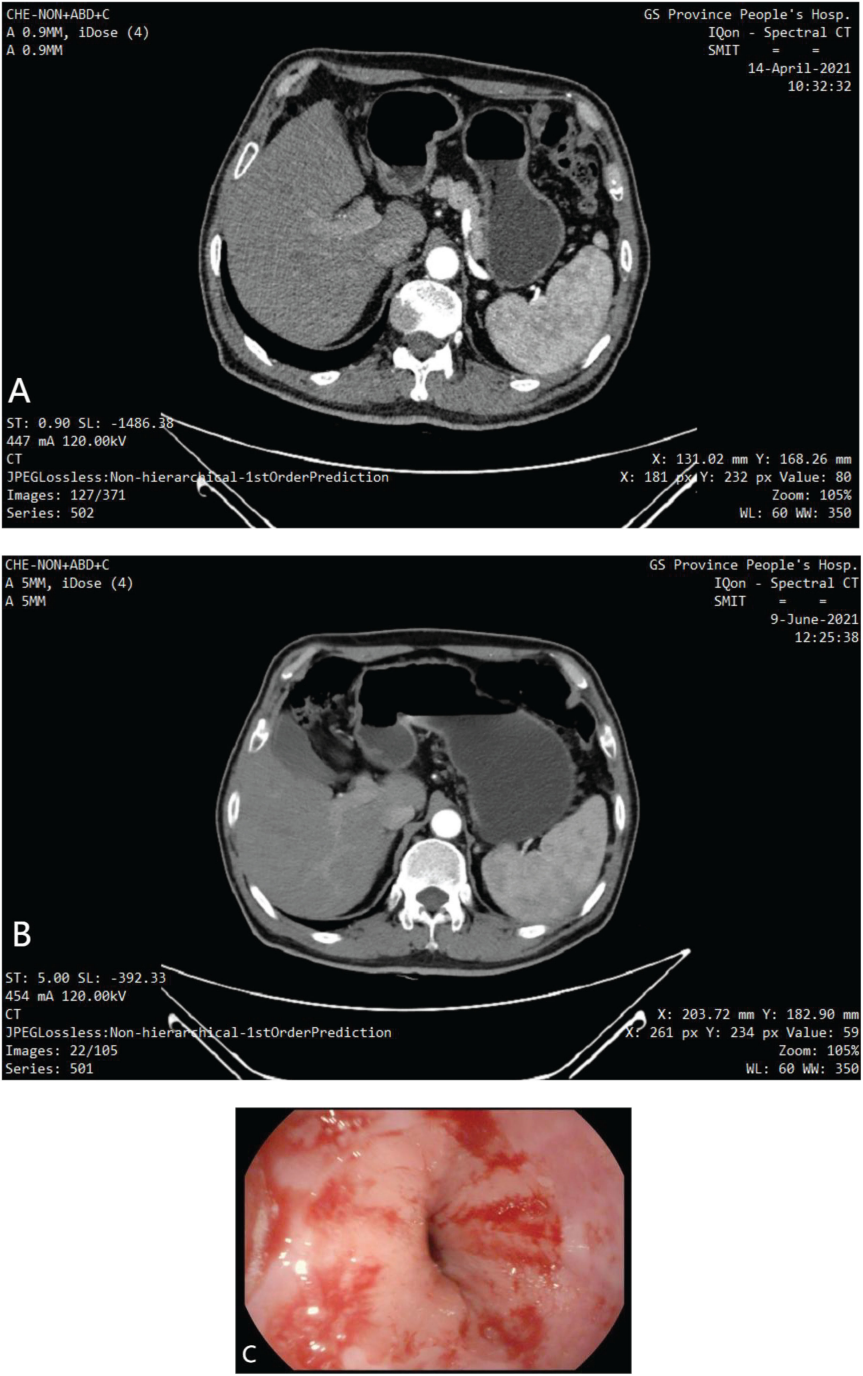
**(A)** Enhanced CT: The gastric wall was thickened and mildly enhanced at the gastric angle and the side of the lesser curvature of the gastric sinus, with a thicker area of about 12 mm, the plasma membrane surface was still smooth, and no obvious enlarged lymph node shadow was seen around. Considered GC (T3N0M0). **(B)** Enhanced CT: The gastric lumen is well filled, no restrictive thickening of the gastric wall, and no abnormal enhancement of the gastric wall in the sinus region. Please combine with the clinical treatment history. **(C)** Electrogastroscopy: The mucosal folds of the gastric body were neatly arranged, and multiple patches of congested erosions were seen.

**Figure 5:**
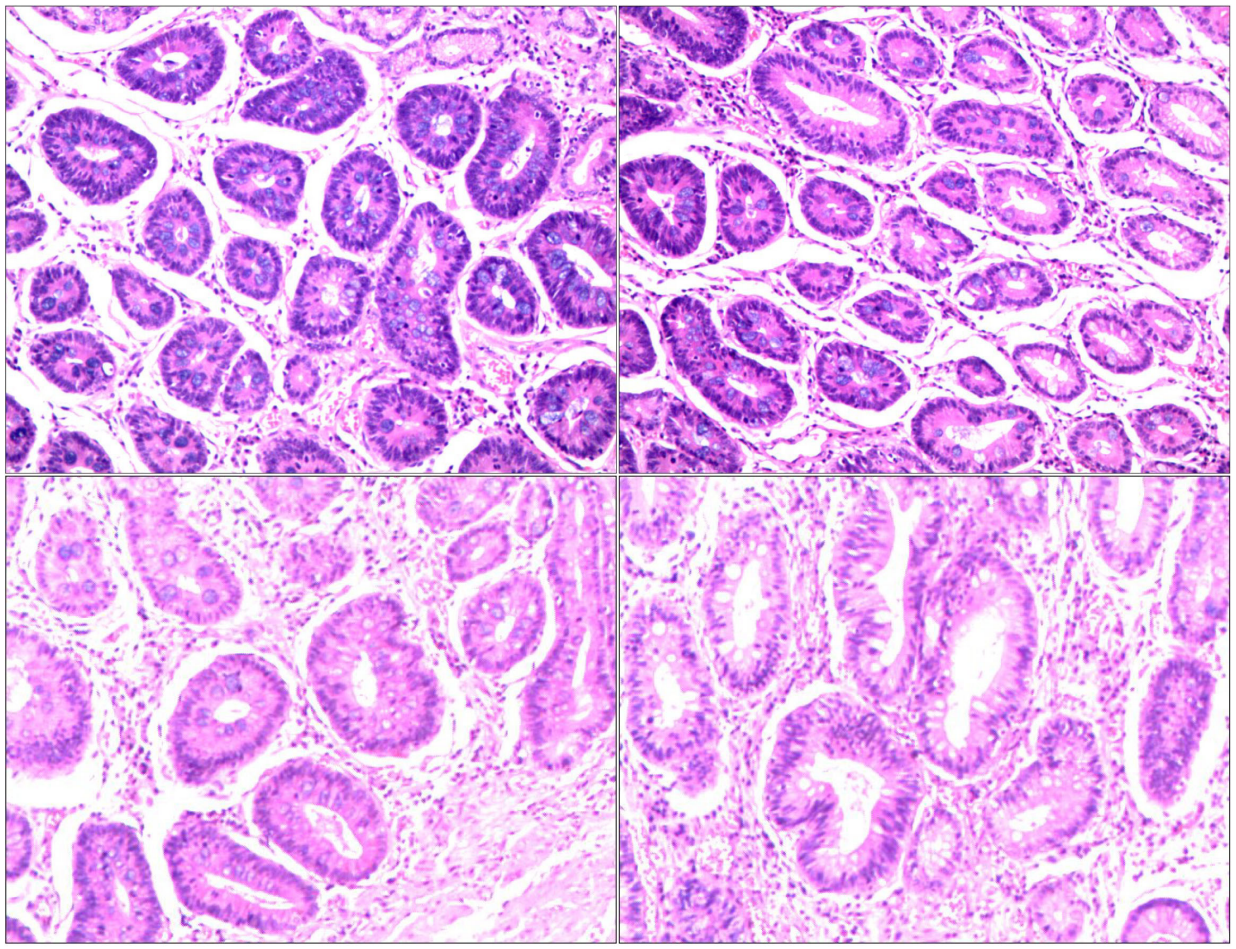
Postoperative pathology: AJCC/CAP pathological assessment criteria after neoadjuvant chemotherapy: TRG: Grade 0 i.e. no cancer cells seen (complete regression) No abnormalities in upper and lower cut ends. 1. stomach (distal): chronic atrophic gastritis with mild intestinalization. Small focal mucosal glands with low-grade intraepithelial neoplasia.2. No significant abnormality in the upper and lower cut ends of the other delivery.3. No metastatic cancer in the lymph nodes; groups 1, 3, 4, 5, 6, 7, 8A.

After the fourth cycle (prior to surgery), the patient was tested for tumor markers, which showed: AFP level of 6.17 ng/mL, CA125 level of 9.5 U/mL, CA199 level of 6.15 U/mL, CEA level of 1.53 ng/mL, CA72-4 level of 2.69 U/mL, a PGI level of 54.3 ng/mL, PGII level of 12.8 ng/mL, and carcinoembryonic antigen level of 0.63 ng/mL. There was no significant change in the tumor markers compared to previous measurements. Meanwhile, the patient was scanned during this period, the tumor were significantly reduced (**Figures 4A–C**), and the clinical stages were cT3N0M0 and No phases, respectively. Subsequently, the patient underwent radical distal gastrectomy and D2 lymph node dissection, followed by Billroth I anastomosis. During the operation, a 2-cm ulcer was found on the greater curvature of the gastric antrum, no regional lymph node enlargement was observed. In addition, no metastatic nodules were found in the liver, parietal peritoneum, mesentery, or pelvic floor. Postoperative pathological diagnosis (**Figure 5**) revealed: AJCC/CAP pathological assessment criteria after neoadjuvant chemotherapy: TRG: Grade 0, no cancer cells seen (complete regression), no abnormalities in upper and lower cut ends. 1: stomach (distal): chronic atrophic gastritis with mild intestinalization. Small focal mucosal glands with low-grade intraepithelial neoplasia. 2: No significant abnormality in the upper and lower cut ends of the other delivery. 3: No metastatic cancer in the lymph nodes; groups 1, 3, 4, 5, 6, 7, 8A. After surgery, the patient received an additional four cycles of SOX trastuzumab and tislelizumab at the same doses as before. Abdominal CT reexamination after cessation of treatment revealed postoperative changes in the gastric malignant tumor. The residual stomach was well filled, and no thickening or enhancement was observed in the residual stomach or the wall of each anastomotic site. No enlarged lymph nodes were observed in the liver or stomach space, and no enlarged lymph nodes were observed in the retroperitoneum. In addition, gastroscopy revealed that the cardia was well opened and closed, no ulcers and masses were observed, and the mucosa was not abnormal. To date, the patient has been followed up for 6 months, and no clinical manifestations have been observed.

## Discussion

As the fifth largest malignant tumor in the world, GC has a high metastasis rate and postoperative local recurrence rate, which is a serious threat to human health (1, 5). In recent years, the rapid development of neoadjuvant chemotherapy, radiotherapy, immunotherapy and molecular targeted therapy provides a variety of options for the treatment of patients with advanced GC (4, 5). Among them, neoadjuvant therapy is more and more widely used in GC. The superiority of preoperative neoadjuvant therapy for GC has also been confirmed by several high-quality Mate analyses. The results show that neoadjuvant therapy can not only effectively reduce the tumor stage and improve the survival rate of patients with advanced GC, but also the effect of multidrug combination chemotherapy is better than that of single drug (16, 17).

Recently, the neoadjuvant regimen of SOX is considered to have great potential and is widely used in clinical practice. As early as ten years ago, G-SOX study showed that SOX was effective in the treatment of unresectable advanced or recurrent GC. The response rate of the 51 patients enrolled in the study was 59%, the disease control rate was 84% and the 1-year survival rate was 71% (18). Feng et al (19) and Satake et al (20) reported earlier the efficacy and safety of SOX neoadjuvant therapy for locally advanced GC, the R0 resection rate is more than 80%, but the pathological complete response rate is only 12.5%. Wang et al (21) compared different neoadjuvant chemotherapy cycles with SOX in the treatment of resectable locally advanced cancer. The results indicate that SOX neoadjuvant chemotherapy has high efficacy and safety in 6-8 cycles and can be widely used in the treatment of locally advanced GC. According to CSCO Guidelines for GC in 2021, SOX has been recommended as a first-line treatment for non-esophagogastric advanced or locally advanced GC (11). However, the existing neoadjuvant chemotherapy regimens do not improve the pathological complete response rate and OS, the prognosis of patients with advanced GC is still very poor and the 5-year survival rate is less than 30% (22–24). Therefore, scholars have never stopped exploring new adjuvant schemes to further improve the pathological complete response rate and OS.

Clinical trial studies with PD-1 antibodies or PD-L1 antibodies have been conducted in a variety of solid tumors (25). Although these PD-1 antibodies are in the trial phase in the above studies, good progress has been made. Gettinger (26) showed an ORR of 23% and a CRR of 7.7% in a study of Nivolumab in NSCLC. In 2014, ESMO published the results of a phase I clinical trial of PD-1 antibodies in advanced metastatic GC showing that 39 patients with a CRR of 22% and an ORR of 30% (27).The KEYNOTE-059 trial explored the efficacy and safety of first-line use of immunotherapy in Her-2 negative advanced GC or gastroesophageal junction adenocarcinoma (28). Cohort 1 results showed an overall population ORR of 11.6% and a median duration of remission of 8.4 months. In the PD-L1-positive subgroup, the ORR reached 15.5% and the overall median duration of remission was 16.3 months, which was better than expected and equally effective in the Asian population (29).The ONO-4538-12, TTRACTION-2 (30) study showed that OS was significantly prolonged in the nabumab group compared to the placebo group (5.26 months vs. 4.14 months, P<0.01) and the 1-year survival rate was 1-fold higher (26.2% vs. 10.9%).

However, immunotherapy is still in the clinical trial stage, and it has become an urgent task to find sensitive biomarkers for PD-1 and PD-L1 inhibitors and to screen the potential beneficiary population. Although immunotherapy is not yet recommended as the treatment of choice, it offers hope for patients with GC who have failed multiple lines of chemotherapy.

## Conclusion

In a word, the results of this study can provide a reference for the neoadjuvant strategy combined with PD-1 inhibitor and trastuzumab on the basis of first-line neoadjuvant regimen for patients with advanced GC that may be unresectable. However, this study has some limitations, and prospective clinical studies with larger samples are needed to verify in the future.

## Data Availability

The original contributions presented in the study are included in the article/supplementary material. Further inquiries can be directed to the corresponding author.
